# New Cosmetic Formulation for the Treatment of Mild to Moderate Infantile Atopic Dermatitis

**DOI:** 10.3390/children6020017

**Published:** 2019-01-29

**Authors:** Raúl de Lucas, Cristina García-Millán, Azahara Pérez-Davó, Esther Moreno, Pedro Redondo

**Affiliations:** 1 Department of Dermatology, Hospital Universitario La Paz, 28046 Madrid, Spain; 2 Dermatology Group Pedro Jaén, 28002 Madrid, Spain; 3 Medical Affairs Department, Cantabria labs, 28043 Madrid, Spain; 4 Department of Dermatology, Clínica Universitaria de Navarra, 31008 Pamplona, Spain; emartero@unav.es (E.M.), predondo@unav.es (P.R.)

**Keywords:** atopic, dermatitis, emollient, moisturizer, epidermal barrier, filaggrin

## Abstract

Atopic dermatitis (AD) is a chronic cutaneous inflammatory disorder, characterized by skin barrier disruption. Dermacare is a new cosmetic formulation, which enhances moisturization, reinforces and repairs the skin barrier, and prevents cutaneous microbiota imbalance. To demonstrate its safety and efficacy, a prospective, open-label, and multicenter study was carried out on patients diagnosed with mild to moderate AD. Transepidermal water loss (TEWL), clinical severity, Desquamation Index, Patient/Investigator Global Assessments, quality of life index, and tolerance were assessed. Adverse events were recorded. Daily application of the new treatment was well tolerated, and adverse events were absent. After 14 days, TEWL showed a 36.7% significant decrease (*p* = 0.035). At the end of the 28-day treatment, the Desquamation Index showed a reduction in 70% of patients; Eczema Area and Severity Index were reduced by 70.4% (*p* = 0.002); and skin irritation showed a significant reduction (*p* = 0.024). Likewise, Patient and Investigator Global Assessments reported a significant improvement in conditions and an overall global worsening when patients restarted their normal treatment. Parent’s Index of Quality of Life Index significantly increased by 36.4% (*p* < 0.05) with Dermacare. In conclusion, a regular use of this new formulation can reduce the risk of relapse and extend the steroid-free treatment periods.

## 1. Introduction

Atopic dermatitis (AD) is a multifactorial chronic inflammatory skin disease characterized by a decreased skin barrier function that is caused by multiple factors, such as environmental and temperature agents, allergens, exogenous irritants, infections, and psychological stress. [1]. 

It is remarkable to highlight that defects in the skin barrier structure, (lack of filaggrin) as well as impairment in functional integrity and reduced ability for self-renewal seem to play a role in triggering both an immune response and nonspecific inflammatory reaction [2].

Atopic children show important immune deregulation, probably due to a change in the cytokine profile synthetized by Th1 and Th2 subpopulations. A decreased number of Th1 lymphocytes is observed in these patients is associated with reduced secretion of interferon (IFN) gamma, which in turn may reduce the activity of natural killer (NK) cells, decrease the number of circulating T lymphocytes, and result in an elevated ratio of CD4+/CD8+ lymphocytes [2]. These cells secrete interleukins (IL-4, IL-5, and IL-13) which induce the production of immunoglobulin E (antibody present in allergies). Also, an exacerbation of itching is the main symptom of atopic dermatitis [3]. As atopic dermatitis involves diverse genetic abnormalities, which affect both the barrier function and the immune system, mutations have been detected in two major groups of genes [4]: Genes encoding structural epithelium proteins and genes encoding elements of the immune system. The family of genes of the epithelial differentiation complex is located in chromosome 1, and one is especially, important filaggrin (FLG), an epidermal protein, with a very important role in the synthesis of keratinocytes [5].

A disrupted skin barrier allows entry of allergens and leads to systemic allergic responses, such as increased IgE. The stratum corneum contributes greatly to skin barrier function [6]. Transepidermal water loss (TEWL) is a non-invasive in vivo measurement of water loss through the stratum corneum to evaluate skin barrier function. Skin in patients with atopic dermatitis always presents microscopic injuries even in remission. These micro-cracks allow the entry of pathogens, allergens, and irritants that contribute to inflammation, infection, and an increase in TEWL. 

Clinically, AD is characterized by intense itching, dry skin, and periodic flares of eczema, usually observed in a characteristic age-dependent distribution with facial, scalp, and extensor involvement in infants and young children, and predominant flexural involvement in older children and adults. The disease usually appears in the first years of life and the location of eczema varies with age [7]. The impact of this disease produces a reduced quality of life, mainly due to a lack of sleep, with low school performance, difficulties in sports, social rejection, and anxiety, and may be the cause of psychological problems in pediatric patients with AD [8]. In several studies based on quality of life, which were carried out in school populations in different countries, over 90% of children have emotional lability that correlate with the severity of the disease [9,10]. Due to its chronic development, the high impact on the quality of life of patients and their families, and its high prevalence in many countries, doctors and patients require adequate strategies to keep patients free of symptoms during remission and improve their quality of life [10].

The prevalence of AD is estimated to be 15–20% in children and 1–3% in adults, and the incidence has increased two to three times over the past decades in industrialized countries [11]. The incidence is similar in both genders, but there are ethnic and geographic differences that suggest the influence of environmental factors in the outcome of the disease. Furthermore, there is a genetic predisposition to suffer from the disease as previously mentioned. The prevalence of AD in children with affected first and second-degree relatives can reach 39% and 19%, respectively [11]. AD usually appears between 3 and 6 months of age so that 60% of the cases appear in the first year [12] and around 85% are diagnosed before the age of five. The prevalence of AD in the general population is difficult to determine, but it is estimated that in advanced industrialized countries, around 20% of children suffer from the disease [11,13]. Most children who suffer from atopic dermatitis do not present symptoms in adulthood, but it is estimated that between 10% and 30% of patients will continue to suffer from it during adult life [14].

Pharmacological treatment must be individually determined for each patient, identifying and correcting triggering factors, determining the extent and location of the lesions, following its evolution. The application of moisturizers reduces dryness and itching and helps prevent flares. Epidermal barrier function is preserved and long periods free of symptoms are achieved with the application of appropriate moisturization twice daily. These results involve less steroid treatment, which is a challenge for dermatologists. Consequently, a new cosmetic treatment has been developed to palliate symptoms and extend the remission period between relapses by Cantabria Labs. It is a lotion formulated with a highly emollient system that enhances skin hydration, as well as the strengthening and repairing the skin barrier function. This emollient formula is supplemented with Pro-Filaggrin Complex, polidocanol, urea, xylitol, and glycyrrhetinic acid. Pro-Filaggrin Complex, which is composed of a glucomannan derived from the yeast, *Candida utilis*, galactoarabinosa, and Niacinamide, reduces typical AD pruritus and its associated scratching. Pro-Filaggrin Complex strengthens the skin barrier by inducing the expression of filaggrin gene. 3% polidocanol and 5% urea act as antipruritic agents. Xylitol and glycyrrhetic acid provide antibacterial and specific protection against biofilm alteration to prevent cutaneous microbiome imbalance, avoid superinfection by *Staphylococcus aureus*, and decrease inflammation, which would lead to exacerbation of erythema and pruritus. We added to the moisturizing treatment a new Syndet cleanser, which is formulated to mitigate pruritus, induce the production of filaggrin, and reduce skin inflammation. This new Syndet is a mild cleanser for daily hygiene of dry, sensitive, and irritated skin, with a 5.5 pH. It cleans in depth and without disturbing the natural balance of the skin, helping in the production of filaggrin, and reducing skin inflammation due to Pro-Filaggrin Complex, Polidocanol, and glycyrrhetic acid. Used daily, both Lotion and Syndet (Cantabria Labs) maintain skin hydration, support barrier function, and reduce TEWL and the desquamation index.

## 2. Study Description

This study was a prospective, pilot, open-label, and multicenter trial. Eight-week clinical studies were conducted to assess the efficacy and safety of this new cosmetic product for the treatment of children diagnosed with mild to moderate atopic dermatitis during remission. The study was carried out by two different pediatric dermatology departments at the La Paz University Hospital (Madrid) and University Clinic of Navarra (Spain). The participating subjects had to have completed a 2-week corticosteroid-wash-out period of 2 weeks, as well as avoid the use of moisturizing creams starting 48 hours before the beginning of the trial.

The primary outcome was to determine the safety of the new cosmetic product, so any adverse events were recorded, and the investigator assessed the severity of skin irritation caused by the product based on erythema, flaking, pruritus, and lichenification. The secondary outcome was to determine the efficacy of the product after the treatment period and to assess the persistent effect of the new cosmetic formulation after a period with ordinary treatment. Efficacy was evaluated by objective and subjective parameters. 

The new cosmetic formulation was applied to patients twice daily for 28 days. One of the times, the lotion had to be applied after bathing with the syndet cleanser. During this period, oral and topical steroids were not allowed. The 19 patients were assessed during 3 visits (day 0, day 14, and day 28). After the first 28 days, treatment was suspended and patients returned to their normal hygiene and moisturizing routines until day 56 when they were assessed a fourth time (visit day 56). 

## 3. Patients and Method

The patients with AD who met eligibility criteria and gave written informed consent were enrolled in this study. The inclusion criteria were children aged 6 months to 3 years with mild-to-moderate AD (according to Scoring Atopic Dermatitis (SCORAD)), with no acute lesions or in remission period, and with informed consent from parents/guardians of children. A total of 19 subjects were recruited. Patients did not use pharmacological treatment during 2 weeks prior to the study or emollient during the 48 h prior to the study, and those presenting any allergy to any of the ingredients in the lotion under study were excluded. Adverse events were recorded throughout the study. Likewise, the dermatologist investigators and the subjects completed efficacy and tolerability assessments at baseline, 14 days, 28 days, and 56 days (V1, V2, V3, and V4, respectively). Clinical assessment through objective parameters, and the recording of adverse events and subjective parameters (Patient/Investigator Global Assessment and Parent’s Index of Quality of Life and Questionnaire for product evaluation by parents, and tolerance by investigator) were developed by dermatologists. 

Transepidermal Water Loss (TEWL) was measured using a Tewameter^®^ TM 300 device (Courage+Khazaka electronic GmbH, Köln, Germany). TEWL is an indispensable parameter for the evaluation of the skin’s water barrier function and even the slightest modification can be identified at an early stage. The device measures the density gradient of water evaporation (g/h/m^2^). Measurements were carried out on the right forearm and right cheek. Desquamation index was measured using the tape-stripping technique with a Corneofix^®^ F 20 device (Courage+Khazaka electronic GmbH). It consists in a special adhesive foil, which collects corneocytes. The number, size, and thickness of the corneocytes indicate the desquamation/hydration level of the stratum corneum. When mounted on the Visioscan^®^ camera (Courage+Khazaka electronic GmbH), the desquamation can be evaluated by its software. The adhesive side of the Corneofix^®^ is applied to the skin area to be evaluated for only a very short time. On removing the tape from the skin, the corneocytes adhere to it. When skin is dehydrated, or damaged, it is characterized by thickened scales and flakes of different sizes. When properly moisturized, however, skin presents regular and small regular corneocytes. Clinical severity was evaluated on a scale of skin irritation, where the investigator established the severity of the pathology based on erythema, scaling, pruritus, and lichenification (0-absent, 1-mild, 2-moderate, 3-severe), and by EASI (Eczema Area and Severity Index), which consists in the measurement of the severity and extent of atopic eczema. The EASI score does not include a grade for dryness or scaling and it includes only inflamed areas. The area score is recorded for four regions of the body (head and neck, trunk, upper limbs, and lower limbs). The area score is the percentage of skin affected by eczema for each body region. The severity score is recorded for each of the four regions of the body. The severity score is the sum of the intensity scores for four signs (redness, thickness, scratching, and lichenification). The average intensity of each sign in each body region is assessed as: None (0), mild (1), moderate (2), and severe (3).

The Parents’ Index of Quality of Life in Atopic Dermatitis (PIQoL-AD) is a Dermatological questionnaire regarding the quality of the child’s life as assessed by the patient’s parents or guardians. It was developed for use in parents of pediatric AD patients, and it is a very useful tool in many studies. The PIQoL-AD score discriminates between different levels of severity and usually correlates with the subjective clinical intensity of atopic dermatitis. The final value of the questionnaire ranges from 0 (highest score) to 28 (lowest score) [15]. Other subjective parameters, such as the Parent Global Assessment (PGA), Investigator Global Assessment (IGA), a questionnaire for the product evaluation by parents and one for assessing product tolerability by the investigator (0: not tolerated - 3: high tolerance), were performed.

### Statistical Analysis

For the analysis of the evolution of quantitative parameters over time, and due that most of them didn’t follow a Gaussian distribution, the non-parametric Friedman test was used as a global signification test. For pair comparisons (interindividual) between baseline and following visits, the non-parametric Wilcoxon test was used. Statistical analysis was performed by SPSS software V24.0, with differences being considered as significant when the significance values obtained from the hypothesis contrast were less than 5% (*p* < 0.05).

## 4. Results

Seventeen of the 19 enrolled patients finished the treatment protocol. Two patients dropped out for reasons not related to the cosmetic treatment. All 19 volunteers were, however, included in the analysis (an intention-to-treat, ITT). The total number of patients included in the study presented an average age of 18 months, having had the disease for an average of 13.4 months and with an average of 3.5 relapses over the preceding 6-month period. No side effects were reported, apart from typical effects the under remission-recurrence cycle of atopic dermatitis.

**Figure 1:**
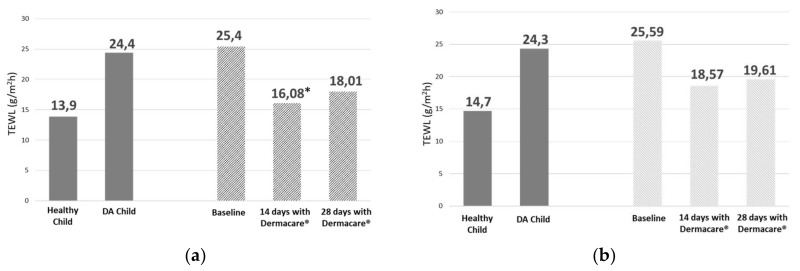
TEWL (transepidermal water loss) values (g/m^2^/h) in healthy children and children with AD (atopic dermatitis) (mean ± SD) [16]; mean TEWL values at baseline and after 14 and 28 days with Dermacare^®^. Evaluations in forearm (**a**) and cheek (**b**); *p = 0.035.

After 14 days of treatment with the new formulation, there was a significant 36.7% decrease (*p* = 0.035) in TEWL on the forearm vs. baseline (falling from 25.4 to 16.08 g/m^2^/h), and a 25.7% reduction in TEWL (statistical trend *p* = 0.061) on the cheek. After 28 days of treatment, TEWL reduction was 29.1% on the forearm and 20.25% in the cheek, not reaching statistical significance (*p* = 0.363 and *p* = 0.334) due to very high intra-individual variability and small sample size (Figure 1). 

**Figure 2:**
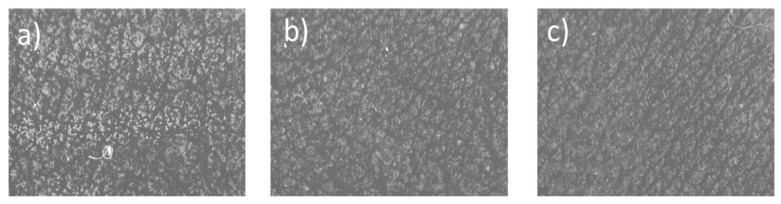
Desquamation index was measured used Corneofix^®^ F 20 device. Images taken by Visioscan^®^ camera. (**a**) Baseline; (**b**) 14 days with Dermacare^®^; (**c**) 28 days with Dermacare^®^.

The analysis according to desquamation index was carried out only on those patients (Figure 2). A reduction in desquamation was observed in 69% of patients at 14 days and in 70% of patients at 28 days. The mean reduction in the desquamation index at 14 days was 30.95% and 21% at 28 days. The global average decrease in the desquamation index in the total population analyzed at both 14 and 28 days, although it did not reach statistical significance (*p* = 0.215 and *p* = 0.208, respectively), shows a decrease, as was previously reported.

**Figure 3:**
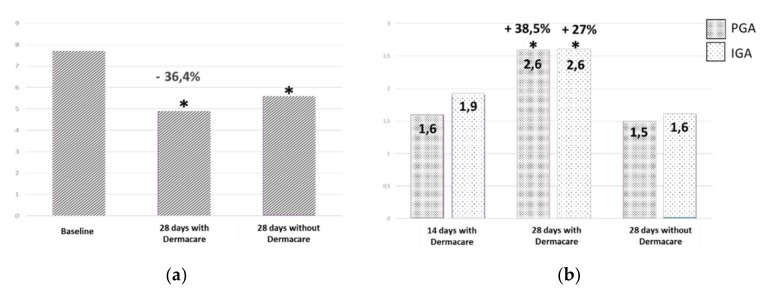
Subjective evaluations: Parents’ Index of quality of Life (**a**), whose interval is between 0 (highest score) and 28 (lowest score) and Patient/Investigator Global Assessments (IGA/PGA) (**b**); *p < 0.05.

The data obtained from the EASI (Eczema Area and Severity Index) used for initial assessment and monitoring during treatment showed a clear and significant improvement over time. The decrease in the severity index score was 45% after 14 days of treatment, representing a significant trend (*p* = 0.066). A significant improvement was achieved after 28 days of treatment (*p* = 0.002), with a decrease of 70.4% in the EASI score vs. baseline. The EASI showed a progressive and significant decrease in the severity of signs of atopic dermatitis, being a parameter of efficacy. The new cosmetic formulation brought about a significant reduction in the severity of basal atopic dermatitis, in only 28 days, with an improvement beginning to appear at 14 days. Investigator global assessment (IGA) reported a significant improvement of 27% (*p* = 0.013) after 28 days of treatment. After the patients returned to their normal treatment, a global worsening of 38.5% was observed by the investigators. Likewise, parent global assessment (PGA) indicated a significant improvement of 38.5% (*p* = 0.05) after 28 days of treatment with the new moisturizer. When the patients returned to the normal treatment, PGA scores worsened by 42.31% in comparison to the PGA score after 28 days of treatment with the cosmetic formulation (Figure 3).

Treatment tolerance was reported as very good from the beginning to the end of the study. The quality of life questionnaire (PI-QoL) was administered at the baseline visit, after 28 days of treatment with the new cosmetic formulation, and the last visit at 28 days after returning to normal treatment. PI-QoL score significantly improved after 28 days of treatment with the new moisturizer cream, passing from 7.7 ± 4.6 to 4.9 ± 3.3, representing a significant improvement of 36.4% (*p* = 0.046) (Figure 2). After completing the period of normal cosmetic treatment, patient quality of life worsened with respect to the score obtained after therapy with this new formulation. The mean score increased to 5.6 ± 3.7, still representing, however, a significant improvement of 27% vs. baseline (*p* = 0.05). The parent product evaluation questionnaire gave a 90% positive product rating. A significant decrease in skin irritation was observed after 28 days of treatment with the formula (*p* = 0.024). The skin irritation value decreased by 55.6% between days 14 and 28.

## 5. Discussion

According to current management guidelines on atopic dermatitis, long-term emollient application, combined with syndet are the basic therapy of atopic dermatitis, but after eczema resolution. Clinical trials show that regular emollient application hydrates the skin and helps to maintain a defensive barrier effect as well as reducing the amount of topical corticosteroids needed for atopic eczema in infants, children, and adult patients. Some studies demonstrate that emollient therapy from birth is a safe and effective approach for atopic dermatitis prevention [17].

The results of trials and long clinical experience have proven that emollients are safe and effective in patients with atopic dermatitis [18,19,20]. Body cream reduces the incidence of flares and the time to flares, reinforcing guidelines that recommend daily emollient therapy as an integral part of maintenance treatment to prevent flares. Although very different emollients have been shown to be beneficial in the prevention of relapses in atopic eczema, we must emphasize that the most important factor in these cases is obtaining adequate ingredients that achieve long remission of the outbreaks.

After 14 days of treatment, a significant increase in cutaneous moisturization was observed due to a decrease in transepidermal water loss vs. baseline. After 28 days of treatment, there was still an improvement in moisturization resulting from a 30% decrease in TEWL, although it was not significant with respect to the baseline. It should be said at this point that statistical significance was difficult to obtain for TEWL values as at 28 days as only 15 patients were analyzed for this parameter vs. 19 at baseline. TEWL values observed during the baseline visit in the study population were similar to those referenced in the literature regarding patients with atopic dermatitis [16,20]. Daily use of both syndet and the new moisturizing formula for 14 and 28 days decreased transepidermal water loss, approaching values considered to be normal in children without AD.

A decrease in desquamation was observed although statistical significance was not reached. This fact is due to two main factors: That the inclusion criteria required patients presenting no active AD lesions and thus the stripping sample was taken from a standardized “healthy skin” area of the patient; and the small sample size. These findings are supported by the reduction in desquamation observed after treatment. After 14 and 28 days of treatment, a 20% and 30% reduction in desquamation was observed in most patients. However, no statistical significance was determined possibly due to the sample size. The EASI severity index showed a significant decrease after just 14 days of treatment, reaching a 70% decrease compared with the baseline. We emphasize that only with 28 days of treatment, there was a significant reduction in the severity of atopic dermatitis symptoms. TEWL, desquamation index, and EASI are objective parameters, which suggest that treatment with this new cosmetic formulation restores skin hydration and decreases the severity of signs present in patients with atopic dermatitis.

The subjective parameters analyzed in the study show that both investigators (IGA) and parents (PGA) reported significative improvement, with an increase in the value given by the investigators of 27%, and 38,4% in the evaluation of the parents/legal guardians of the patient, and both improvements were statistically significant. After returning to the normal treatment for 28 days (visit 4), IGA reported a remarkable reduction of 38.5 % compared to the one evaluated after 28 days with the new formula treatment (visit 3). The PGA reduction was 42.3%. After finishing the period with usual cosmetic treatment, the quality of life of the patient got worse with respect to the score obtained after therapy with both syndet and the new moisturizer. The mean score increased to 5.6 + 3.7. However, regarding the score of the baseline, a significant improvement of 27% was obtained (*p* = 0.05). The worsening observed after discontinuing treatment (returned to values to achieved at 14 days) allows us to confirm that to maintain the improvement obtained with this new formula, this treatment must be continued for a longer period of time. In addition, there was a positive correlation between the opinion of the investigators and the parent regarding the improvement achieved with the treatment and in the worsening after stopping it. Quality of life showed a significant improvement after 28 days of treatment. After discontinuing the new cosmetic formulation and returning to ordinary treatments, a slight, but significant worsening of the quality of life was observed. These significant differences show the importance of maintenance treatment with specific moisturizers to improve quality of life of patients with atopic dermatitis. We must highlight that the new cosmetic formulation showed a very good tolerance and no adverse effects were reported during treatment.

## 6. Conclusions

Despite the high variability of the objective data and the small study sample size due to the pilot nature of the study and dropout of the population at visit 2 (28 days with treatment), cutaneous moisturization of patients with AD improved with this new formulation treatment. This affirmation is supported by the decrease in the values of transepidermal water loss (TEWL) and the reduction in skin flaking. Some authors report that treatment with an adequate moisturizer is beneficial for the dry skin of patients with AD, especially during the dry, cold season [17]. Treatment with our new cosmetic formulation restores the skin barrier by increasing the cutaneous hydration observed as a result of the reduction of cutaneous desquamation and transepidermal water loss. We obtained cutaneous hydration values similar to those described by Choi SJ et al. [20] in children without atopic dermatitis. The restoration of the skin barrier promotes optimal hydration and prevents the penetration of external agents, such as allergens or microorganisms, that cause worsening of atopy symptoms. The positive results obtained in the objective parameters were supported by the improvement reported by both the investigators and the parents of the patients, and by the improvement in quality of life.

The fact observed that improvement during the treatment with the new formula and the worsening when it was discontinued suggested that maintenance treatment during remission can increases time between relapses. Likewise, this formulation could give support to decreasing the number of times in which the use of corticosteroids is necessary and, consequently, the possible occurrence of side effects as a result of their use. However, it is believed that further studies are warranted to confirm the hypothesis of prolonging the corticoid-free period to avoid skin dystrophy typical of corticoid use.
